# Association between denture use, chewing ability, and all-cause mortality in middle-aged and older adults who exercised regularly in Korea

**DOI:** 10.1038/s41598-021-85440-x

**Published:** 2021-03-15

**Authors:** Jong-Hwa Jang, Ji-Liang Kim, Jae-Hyun Kim

**Affiliations:** 1 Department of Dental Hygiene, College of Health Science, Dankook University, Cheonan, 31116 Korea; 2 Department of Public Health, General Graduate School of Dankook University, Cheonan, 31116 Korea; 3 Department of Health Administration, College of Health Science, Dankook University, Cheonan, 31116 Korea

**Keywords:** Biomarkers, Diseases, Health care, Medical research, Risk factors

## Abstract

Herein, using data from the Korean Longitudinal Study of Aging (2006–2018), we evaluated denture use and chewing ability to determine the status of oral health in middle-aged adults who exercised regularly; further, we investigated the relationship of oral health with all-cause mortality. From the basic survey conducted in 2006, we interviewed 10,254 participants who were followed up until death. The participants were grouped based on regular exercise into REG (n = 3921) and non-REG (n = 6290) groups. The mortality rate was higher in the non-REG group than in the REG group (35.8% versus 26.9%; *p* < 0.001). The mortality rate was higher in denture users (versus non-denture users), non-drinkers (versus alcohol drinkers), and those on medical aid (versus national health insurance). The mortality rate was higher in participants with poor masticatory ability, lower education level, and poor subjective health perception (*p* < 0.001). Denture use and masticatory discomfort were not significant risk factors for mortality in the non-REG group (*p* > 0.05). In conclusion, masticatory discomfort was a risk factor for increased mortality in middle-aged Korean adults who exercised regularly, at least once a week. Thus, assessment of masticatory ability could be a useful indicator of life expectancy in middle-aged adults.

## Introduction

Owing to an increase in the average life expectancy as a result of advancements in science and medicine, life after middle age has become an important stage in the life cycle of human beings. There is an increase in the desire for a better quality of life after middle age^1^_._

Oral health problems can deteriorate an individual’s quality of life, with detrimental effects on their well-being^2^; thus, oral health can be an important index for health evaluation^3^. Oral health has been closely associated with general health, including physical strength^4^; in addition, it has been associated with physical and cognitive decline^5^. In the elderly, tooth loss, an oral health problem for several centuries, is usually caused by chronic conditions, such as dental caries or periodontal disease^5^. Moreover, it is considered as an early indicator of physical and cognitive decline as well as senility due to advancing age^6^. Tooth loss, which is a risk factor for the declining quality of life, is a life-long source of financial burden owing to the need for dental treatment^7,8^. An association between tooth loss, which is the biggest cause of masticatory dysfunction, and cardiovascular disease has been reported^9,10^. Several previous studies in larger cohorts, with follow-up periods of 1–56 years, have reported an association of tooth loss with morbidity, disability-onset, and early mortality^11–16^. As previously reported, individuals with fewer teeth or poor masticatory ability, due to non-replacement of teeth, are at a higher risk of developing cognitive impairment, such as dementia, than those with more than 20 teeth and good masticatory ability^17,18^. Numerous studies have reported that masticatory discomfort, caused by tooth loss, is a risk factor for increased mortality^11,13,14,16^. Masticatory discomfort directly influences individual, physical, psychological, and oral health^19^.

Maintaining physical fitness has a positive effect on various biological functions in oral mucosa^20^. For the development of oral health programs focused on increasing the life expectancy in middle-aged/older adults, it is necessary to investigate whether the chewing ability is an early risk factor, associated with mortality, in middle-aged older adults who exercise regularly^2^. Currently, there is a lack of longitudinal studies focusing on the association of denture use and chewing ability with mortality in middle-aged older adults who exercise regularly. According to a previous study^12^, cohort data from follow-up studies can be useful for identifying oral health as a risk factor for mortality. We hypothesized that the use of dentures and chewing ability may be associated with all-cause mortality in adults aged ≥ 45 years, who exercised regularly, at least once a week.

In this study, using data from the Korean Longitudinal Study of Aging (KLoSA), we aimed to investigate denture use and chewing ability, to determine the oral health, in middle-aged and older adults who exercised regularly. Further, we investigated the relationship of oral health with all-cause mortality.

## Methods

### Study sample and design

**Figure 1:**
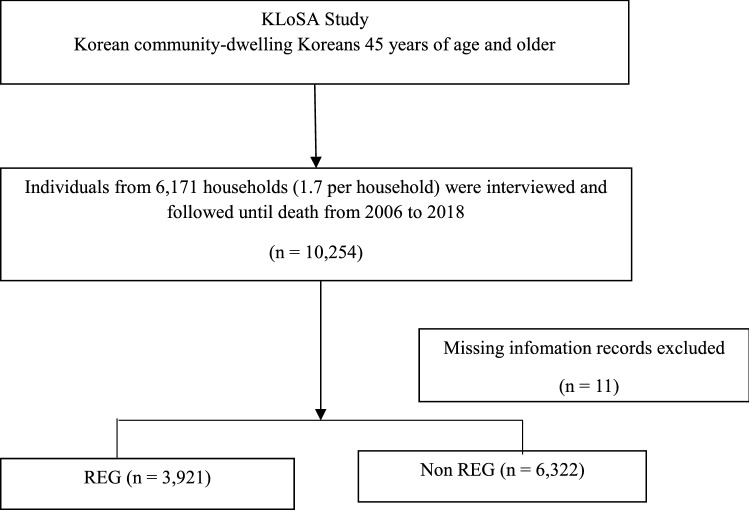
Flow chart of participants included in this study.

In this study, we used KLoSA data collected between 2006 and 2018. KLoSA, a cohort study by the Korea Employment Information Service, was conducted to build datasets needed for making policies to address trends related to population aging^21^. The Koreans, aged ≥ 45 years, living in local communities, provided secondary data, without personal information, on the KLoSA website (https://survey.keis.or.kr/eng/klosa/klosa01.jsp). Random, multi-level stratified probability sampling, based on geographic area and type of residence, was done to select participants nationwide. From the first basic survey conducted in 2006, 10,254 participants, from 6,171 households (1.7 people per household) were investigated and followed up until death; among them, 3921 participants who claimed to exercise regularly, at least once a week, were assigned to the exercise group (REG), and 6322 participants who did not exercise were assigned to the non-exercise control group (non-REG). Eleven participants, with missing information from the 2006 survey were not included in the study (Fig. 1).

### Variables and measurements

Trained investigators obtained consent from individuals who wished to voluntarily participate in the study; they conducted face-to-face interviews, using a computer-based personal interview software, according to the research protocol of the KLoSA. Denture use, an independent variable, was measured by asking the participant whether he/she wore dentures on a regular basis; their responses were recorded as “yes” or “no.” Self-assessed chewing ability was evaluated by asking the participant if he/she could chew on hard foods, such as apples and meat, without difficulty while wearing dentures. The responses were recorded as “good,” “usually,” or “bad.” All-cause mortality (n = 1941), which was an outcome variable, was determined using death certificates and medical examiners’ reports at follow-up. All-cause mortality was determined according to the International Classification of Disease, 10th version^22^. The socioeconomic and demographic characteristics (n = 6) as well as the health status and behavioral factors (n = 3) were used as control covariate variables. Age (a demographic and sociological characteristic) was divided into the following four categories: 45–54 years, 55–64 years, 65–74-years, and 75 years. The level of education was classified into four groups: elementary school or lower, middle school, high school, and university or higher. Sex was identified as either male or female. The marital status was classified as married, divorced or separated, and unmarried. Work restrictions, due to health conditions, were divided into two groups: “yes” and “no.” The participants’ insurance was categorized into national health insurance (NHI) and medical aid (MA). Drinking (a health condition and behavioral factor) was assessed with a “yes” or “no” question. The number of chronic diseases affecting an individual was measured by counting the diseases; they included hypertension, diabetes, cancer, chronic obstructive pulmonary disease, liver disease, cardiovascular disease, cerebrovascular disease, mental disease, and arthritis. Subjective health perception was graded as “very good,” “good,” “neutral,” “bad,” or “very bad.”

### Ethical consideration

All procedures performed in our study that involved human participants were in accordance with the ethical standards of the institutional and/or national research committee and with the 1964 Helsinki declaration and its later amendments or comparable ethical standards (DKU202008013-UE002). Informed consent was obtained from all individual participants included in the study.

### Statistical analysis

The chi-square test, log-rank test, and Cox proportional hazards models were used to analyze the association between the chewing ability and all-cause mortality. Using the Cox proportional hazard model, the adjusted hazard ratio (aHR) and 95% confidence intervals (CI) were calculated to assess the effects of denture use and chewing ability on mortality. Survival time was the outcome variable, which was measured as the time interval between the date of enrollment and the date of death or censoring (up to 12 years). Kaplan–Meier survival analysis and the log-rank test were used to evaluate the cumulative incidence of all-cause mortality, with respect to chewing ability and denture use. A *p *value of < 0.05 was considered significant for all two-tailed tests. Statistical analysis was performed using SAS software (Version 9.4; SAS Institute).

## Results

**Figure 2:**
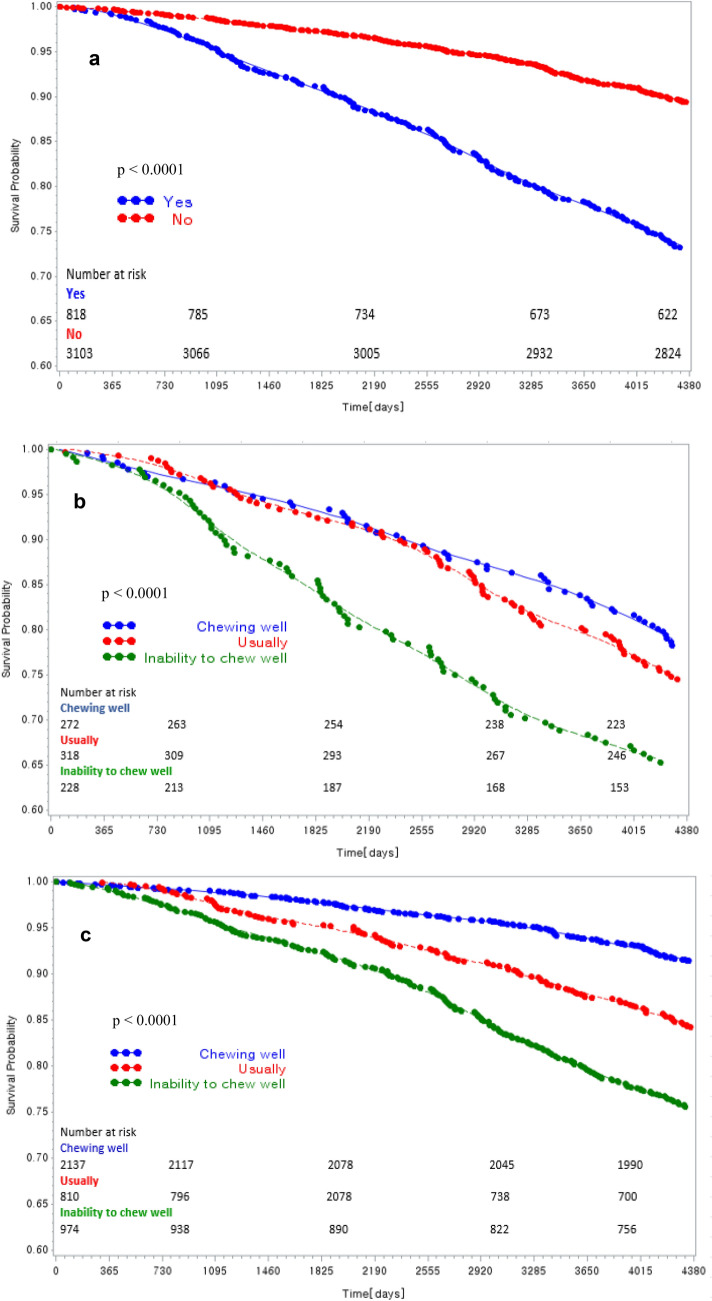
Kaplan–Meier plot showing the mortality, by the denture use and chewing abilities, in the regular exercise group. The Kaplan–Meier curve is an estimator of the survival function. The Kaplan–Meier curve is the visual representation of this function and shows the probability of an event at a given time interval. The *p* value is for the log-rank test. (**a**) The cumulative mortality based on the denture use (**b**) The cumulative mortality based on the chewing ability of denture users (**c**) The cumulative mortality rate based on the chewing ability of denture non-users.

A total of 1,941 participants (18.95%) died; the mortality rate of denture wearers, among the middle-aged/older Korean participants who were followed up for 12 years, was 31.4% (813). The mortality rate was higher in the non-REG group than in the REG group (35.8% versus 26.9%; *p* < 0.001; Table 1); increment in the mortality rate was observed with an increase in masticatory discomfort (*p* < 0.01). The mortality rate was higher in those who used dentures (versus non-denture users), in older participants (versus younger), men (versus women), unmarried and divorced/separated participants (versus married), those with work restrictions (versus no work restrictions), those on the MA (versus NHI), and in non-drinkers (versus alcohol drinkers). The mortality rate was higher in participants with poor masticatory ability and lower education level and subjective health perception (*p* < 0.001). The Kaplan–Meier survival chart showing the cumulative survival rate, regarding denture use and chewing ability of the REG group, is presented in Fig. 2.

**Table 1:** Characteristics and univariate analysis of factors influencing mortality according to regular exercise at baseline.

	REG (n = 3921)		*p* value	Non-REG (n = 6290)		*p* value
	Dead	Alive		Dead	Alive	
**Denture use**						
Yes	220 (26.9)	598 (73.1)	< 0.001	593 (35.8)	1062 (64.2)	< 0.001
No	331 (10.7)	2772 (89.3)		797 (17.2)	3838 (82.8)	
**Chewing abilities wearing denture**						
Good	59 (21.7)	213 (78.3)	0.004	133 (31.4)	290 (68.6)	0.012
Usually	82 (25.8)	236 (74.2)		200 (34.4)	381 (65.6)	
Bad	79 (34.7)	149 (65.4)		260 (39.9)	391 (60.1)	
**Chewing abilities without denture**						
Good	183(8.6)	1954 (91.4)	< 0.001	267 (10.8)	2212 (89.2)	< 0.001
Usually	129 (15.9)	681 (84.1)		366 (22.3)	1277 (77.7)	
Bad	239 (24.5)	735 (75.5)		757 (34.9)	1411 (65.1)	
**Age**						
≤ 54	70 (4.8)	1381 (95.2)	< 0.001	96 (5.2)	1738 (94.8)	< 0.001
55–64	113 (9.7)	1051 (90.3)		178 (11.0)	1446 (89.0)	
65–74	222 (22.9)	748 (77.1)		462 (27.2)	1238 (72.8)	
> 75	146 (43.5)	190 (56.6)		654 (57.8)	478 (42.2)	
**Education**						
≤ Elementary school	277 (22.4)	962 (77.6)	< 0.001	1059 (29.8)	2498 (70.2)	< 0.001
Middle school	80 (11.4)	620 (88.6)		131 (13.7)	824 (86.3)	
High school	125 (9.5)	1188 (90.5)		151 (10.9)	1239 (89.1)	
College and above	69 (10.3)	600 (89.7)		49 (12.6)	339 (87.4)	
**Sex**						
Male	346 (18.2)	1555 (81.8)	< 0.001	651 (25.6)	1893 (74.4)	< 0.001
Female	205 (10.2)	1815 (89.9)		739 (19.7)	3007 (80.3)	
**Marital status**						
Married	410 (12.4)	2894 (87.6)	< 0.001	816 (17.6)	3828 (82.4)	< 0.001
Separated, divorced	139 (23.2)	461 (76.8)		563 (35.8)	1008 (64.2)	
Unmarried	2 (11.8)	15 (88.2)		11 (14.7)	64 (85.3)	
**Working restriction**						
Yes	259 (24.6)	796 (75.5)	< 0.001	836 (34.3)	1605 (65.8)	< 0.001
No	292 (10.2)	2574 (89.8)		554 (14.4)	3295 (85.6)	
**Health insurance**						
National health insurance	506 (13.5)	3235 (86.5)	< 0.001	1237 (21.2)	4594 (78.8)	< 0.001
Medical aid	45 (25.0)	135 (75.0)		153 (33.3)	306 (66.7)	
**Alcohol consumption**						
Yes	210 (12.8)	1429 (87.2)	0.058	406 (18.8)	1757 (81.2)	< 0.001
No	341 (14.9)	1941 (85.1)		984 (23.8)	3143 (76.2)	
Number of chronic diseases*						
0	181 (8.8)	1876 (91.2)	< 0.001	513 (16.0)	2698 (84.0)	< 0.001
1	193 (17.3)	924 (82.7)		446 (23.2)	1396 (75.8)	
≥ 2	177 (23.7)	570 (76.3)		431 (34.8)	806 (65.2)	
**Self-rated health**						
Very good	15 (7.1)	197 (92.9)	< 0.001	14 (9.7)	130 (90.3)	< 0.001
Good	129 (8.1)	1455 (91.9)		221 (11.6)	1687 (88.4)	
Moderate	169 (14.0)	1040 (86.0)		363 (18.3)	1624 (81.7)	
Bad	186 (23.9)	593 (76.1)		529 (31.3)	1164 (68.8)	
Very bad	52 (38.0)	85 (62.0)		263 (47.1)	295 (52.9)	

*REG* Regular exercise group, *p* values were calculated by chi-squared tests.

*Hypertension, diabetes, cancer, chronic obstructive pulmonary disease, liver disease, cardiovascular disease, cerebrovascular disease, arthritis; The data are presented as n (%).

### Association between denture use and all-cause mortality

The association between denture use, chewing ability, and mortality was analyzed after adjusting for age, education, sex, marital status, work restriction, health insurance, alcohol consumption, number of chronic diseases, and self-rated health (Table 2). The aHR of the REG group with regular denture use was 1.29 (95% CI: 1.07–1.55; *p* = 0.007); the aHR after adjusting for chewing ability was 1.30 (95% CI: 1.05–1.60). No significant association was observed between denture use and mortality in the non-REG group (*p* = 0.860).

**Table 2:** Adjusted hazard ratios and 95% confidence intervals for the associations between denture use and all-cause mortality in middle aged and older adults from the Korean Longitudinal Study of Aging 2006–2018.

	Model 1			Model 2		
	aHR	95% CI	*p* value	aHR	95% CI	*p* value
**REG (n = 3921)**						
Denture use						
Yes	1.291	1.07–1.55	0.007	1.297	1.05–1.60	0.016
No	1.000			1.000		
Chewing abilities						
Good				1.000		
Usually				1.002	0.79–1.28	0.990
Bad				0.990	0.77–1.28	0.941
**Non-REG (n = 6290)**						
Denture use						
Yes	1.029	0.92–1.15	0.624	1.011	0.89–1.15	0.860
No	1.000			1.000		
Chewing abilities						
Good				1.000		
Usually				1.048	0.88–1.25	0.598
Bad				1.074	0.90–1.28	0.429

*aHR* Adjusted hazard ratio, *CI* Confidence interval, *p* value using Cox model, *REG* Regular exercise group. All models were adjusted for age, education, sex, marital status, working restriction, health insurances, alcohol consumption, number of chronic diseases, and self-rated health.

### Associations between chewing ability and all-cause mortality with respect to denture use

The association between chewing ability and mortality in denture wearers was analyzed using the Cox model after adjusting for age, education, sex, marital status, work restriction, health insurance, alcohol consumption, number of chronic diseases, and self-rated health (Table 3). Among the denture wearers of the REG group, chewing ability was associated with mortality, with a respective aHR of 1.20 and 1.54, in the subgroups “usually” versus “good” (95% CI: 0.85–1.69) and “bad” versus “good” (95% CI: 1.08–2.19). The aHR, after adjusting for the chewing ability in non-denture wearers, was 1.28 in participants who responded “usually” (versus good; 95% CI: 0.88–1.86) and 1.62 in those who responded “bad” (versus good; 95% CI: 1.12–2.36). The relationship between chewing ability and mortality was not significantly different in the non-REG group (*p* = 0.871).

**Table 3:** Adjusted hazard ratios and 95% confidence intervals for the associations between chewing abilities and all-cause mortality in the denture wearing group from the Korean Longitudinal Study of Aging 2006–2018.

	Model 1			Model 2		
	aHR	95% CI	*p* value	aHR	95% CI	*p* value
**REG (n = 3921)**						
Chewing abilities with denture						
Good	1.000			1.000		
Usually	1.201	0.85–1.69	0.295	1.278	0.88–1.86	0.199
Bad	1.536	1.08–2.19	0.017	1.623	1.12–2.36	0.011
Chewing abilities without denture						
Good				1.000		
Usually				0.724	0.38–1.38	0.325
Bad				0.747	0.43–1.30	0.299
**Non-REG (n = 6290)**						
Chewing abilities with denture						
Good	1.000			1.000		
Usually	1.015	0.81–1.27	0.896	1.027	0.81–1.31	0.826
Bad	0.988	0.79–1.23	0.912	1.019	0.81–1.28	0.871
Chewing abilities without denture						
Good				1.000		
Usually				0.853	0.55–1.33	0.485
Bad				0.803	0.54–1.19	0.274

*aHR* Adjusted hazard ratio, *CI* Confidence interval, *p* value using Cox model, *REG* Regular exercise group. All models were adjusted for age, education, sex, marital status, working restriction, health insurances, alcohol consumption, number of chronic diseases, and self-rated health.

## Discussion

We hypothesized that the use of dentures and chewing ability may be associated with all-cause mortality in middle-aged and older adults, who exercised regularly, at least once a week. Therefore, we assessed the association between all-cause mortality and oral health in adult Koreans aged ≥ 45 years using data from a long-term follow-up survey conducted by the KLoSA.

Exercise is essential for the promotion and maintenance of good health^4^. However, despite regular exercise, oral health may be a risk factor for mortality in middle-aged and older adults^2^. To the best of our knowledge, no study has identified oral health characteristics as a risk factor for mortality in middle-aged/older adults who exercise regularly^11,16,23–26^.

The maintenance of 20 or more natural teeth allows proper mastication and, therefore, is considered an index of oral health^27,28^. The number of elderly Korean individuals, with more than 20 natural teeth, increased from 45.8% in 2010 to 53.7% in 2015. However, the rate of masticatory discomfort decreased only slightly from 44.3% in 2010 to 42.9% in 2017^29^. Thus, it is expected that oral health problems in the elderly will continue, despite an increase in the number of teeth retained in the oral cavity.

In this study, the mortality rate was lower among denture wearers than non-denture wearers (REG group, 26.9%; non-REG group, 35.8%). It is reported that oral health is significantly related to age, race, family income and wealth, smoking, metabolic syndromes, and physical activity^30^.

In our study, age was strongly associated with all-cause mortality. In particular, a strong association with all-cause mortality was observed in the elderly participants (aged ≥ 75 years) of the non-REG group (57.8%): it was higher than that in the REG group (43.5%). The mortality rate was the highest in participants with elementary school or lower education level (REG group, 22.4%; non-REG group, 29.88%). These results were similar to the findings of Shin and Jung^19^ who reported that tooth loss and masticatory discomfort in the elderly were associated with lower education levels.

In the REG group, men (18.2%) had a higher mortality rate than women (10.2%). According to a recent study^26^, the masticatory disorder was an important risk factor for male mortality and predictor of life expectancy in elderly men. Divorced or separated elderly individuals (23.2%) had a higher mortality rate than their married counterparts (12.4%). The mortality rate in elderly individuals with work restrictions (24.6%) was higher than that in those without restrictions (10.2%). The mortality rate was higher in those who used MA (25.0%) than those who used the NHI (13.5%). The difference in the mortality rate was statistically significant between drinkers (18.8%) and non-drinkers (23.8%) in the non-REG group. The mortality rate increased with chronic conditions, such as hypertension, diabetes, cancer, chronic obstructive pulmonary disease, liver disease, cardiovascular disease, cerebrovascular disease, and arthritis. The mortality rate in individuals with more than two chronic conditions in the REG and the non-REG group was 23.7% and 34.8%, respectively. The mortality rate increased in individuals with the poor subjective perception of health; 38.0% and 47.1% participants in the REG and non-REG group, respectively, answered that their health was “very bad.” Overall, the mortality risk was higher in participants with poorer sociodemographic characteristics and health behavior. In particular, the risk of mortality was higher in the non-REG group than in the REG group, suggesting that exercise is important for improvement in health^31^.

In the REG group, the model was adjusted for age, education, sex, marital status, working restriction, health insurances, alcohol consumption, number of chronic diseases, and self-rated health perception to investigate the effect of masticatory ability on the risk of mortality. Chewing ability was not a significant risk factor for mortality; however, the mortality risk in denture wearers was high (aHR = 1.30; *p* = 0.016). In previous studies^16^, the mortality rate was 1.3-fold higher in participants with dental prostheses or edentulous jaws than those with more than 20 natural teeth. Furthermore, it was reported that individuals with inadequate masticatory ability, including denture wearers, had a 15% or higher risk of mortality than those with good chewing function^16^, which was similar to that observed in this study. However, in a prospective cohort study, including elderly individuals in China^14^, Yuan et al. reported a decrease in mortality (aHR = 0.81; 95% CI: 0.77–0.84) with denture use. The difference may be attributed to the higher number of missing teeth in denture wearers than in non-denture wearers in their study. In contrast, despite the use of dentures, the risk of mortality increased in denture wearers if the chewing ability was poor (aHR = 1.54; *p* = 0.017); the aHR was 1.62 (*p* = 0.011) after adjusting for chewing ability in non-denture wearers. The analysis of the cumulative incidence of all-cause mortality, according to chewing ability and denture use, with Kaplan–Meier survival analysis and log-rank test revealed a significant association between denture use and masticatory discomfort (*p* < 0.001). This finding is consistent with previously reported findings^12–14,23,26,32,33^. However, denture use and masticatory disorder were not significant risk factors for increased mortality in the non-REG group (*p* > 0.01). This observation supports the hypothesis that although regular exercise is an important factor affecting overall health, individuals who exercise regularly but have poor oral health may have a relatively high risk of mortality. This study found that denture use and masticatory discomfort were significant risk factors for higher all-cause mortality in middle-aged/older adults, even in those who exercised more than once a week.

This study has some limitations. The KLoSA, whose panel data was used here, was a longitudinal study, which was conducted between 2006 and 2018^21^. To assess oral health, we only measured chewing ability and denture use through interview surveys. Therefore, all risk factors related to oral health status or behavior, that could influence mortality, were not analyzed. Although several studies have focused on the relationship between oral health and death, a comparative analysis of the results was difficult because there were no prior studies in middle-aged and elderly people who exercised regularly. There could be a possibility of selection bias during the enrollment process, but we calculated the cumulative incidence in models adjusted for psychosocial and health-related factors. Despite these limitations, this was the first cohort study to demonstrate the relationship between oral health and mortality, with respect to denture use and subjective chewing ability, in middle-aged and older Korean adults. Oral examinations have been conducted in the KLoSA since 2019. Thus, we believe that a study that focuses on the relationship between mortality and various oral health-related indicators, such as tooth number, should be conducted in the future for more conclusive results. Furthermore, the association between masticatory disorder and mortality needs to be assessed through community-based cohort studies, after controlling for nutritional problems caused by systemic diseases^34–37^, and psychological factors^38^.

In summary, although there was no significant difference in middle-aged and older adults who did not exercise regularly, we found that mastication disorder was an important risk factor for mortality in middle-aged and older adults who exercised regularly and used dentures (for missing teeth). The degree and ease with which hard foods, such as apples and meat, can be chewed may serve as an indicator of the masticatory disorder. Therefore, assessing masticatory ability may be a useful indicator of life expectancy in middle-aged and older adults who exercise regularly. We hope that our findings will contribute to the development of oral health programs focused on improving masticatory ability and regular exercise to increase the life expectancy in middle-aged and older adults.

## Data Availability

The data of the KloSA are publicly available through the KLoSA website (https://survey.keis.or.kr/klosa/klosa01.jsp). The data generated during and/or analyzed the current study are available from the corresponding author on a reasonable request.
